# A unique feature of swine ANP32A provides susceptibility to avian influenza virus infection in pigs

**DOI:** 10.1371/journal.ppat.1008330

**Published:** 2020-02-21

**Authors:** Haili Zhang, Hongxin Li, Wenqiang Wang, Yujie Wang, Guan-Zhu Han, Hualan Chen, Xiaojun Wang

**Affiliations:** 1 State Key Laboratory of Veterinary Biotechnology, Harbin Veterinary Research Institute, The Chinese Academy of Agricultural Sciences, Harbin, China; 2 College of Life Sciences, Nanjing Normal University, Nanjing, Jiangsu, China; Oxford University, UNITED KINGDOM

## Abstract

Both the replication and transcription of the influenza virus are catalyzed by the viral polymerase complex. The polymerases of most avian influenza A viruses have poor performance in mammalian cells, which is considered to be one of the important species barriers. Pigs have been long considered as important intermediate hosts for interspecies transmission of the avian influenza virus, because of their susceptibility to infection with both avian and mammalian influenza viruses. However, the molecular basis of influenza polymerase adaptation in pigs remains largely unknown. ANP32A and ANP32B proteins have been identified as playing fundamental roles in influenza virus replication and host range determination. In this study, we found that swine ANP32A (swANP32A), unlike swine ANP32B or other mammalian ANP32A or B, shows stronger supporting activity to avian viral polymerase. Knockout of ANP32A in pig cells PK15 dramatically reduced avian influenza polymerase activity and viral infectivity, suggesting a unique feature of swANP32A in supporting avian influenza viral polymerase. This species-specific activity is mapped to two key sites, 106V and 156S, in swANP32A. Interestingly, the amino acid 106V is unique to pigs among all the vertebrate species studied, and when combined with 156S, exhibits positive epistasis in pigs. Mutation of 106V and 156S to the signature found in ANP32As from other mammalian species weakened the interaction between swANP32A and chicken viral polymerase, and reduced polymerase activity. Understanding the molecular basis of ANP32 proteins may help to discover new antiviral targets and design avian influenza resistant genome edited pigs.

## Author summary

The pig is considered to be a “mixing vessel” for influenza viruses because it can be infected by both human and avian influenza viruses. This mixing of viruses could potentially produce novel recombinant strains that are more adaptable to humans or other mammals. The permissive mechanism that allows pig cells to be infected with avian influenza virus is largely unknown. Here we reveal that the pig cellular protein ANP32A has a unique 106V/156S signature, different from that of ANP32A proteins from other mammals, enabling the protein to bind strongly to avian influenza polymerase at the post-entry step, and promoting avian virus replication. This species-specific 106V/156S epistasis of swANP32A likely determines the susceptibility of pigs to avian influenza infection. Our findings provide novel insights into the molecular basis of interspecies transmission of avian IAV between chickens and pigs.

## Introduction

Influenza A viruses (IAVs) are highly infectious respiratory pathogens that can infect many species, posing a great threat to both veterinary and human public health. Aquatic birds are considered to be the largest natural reservoir for IAVs. The four influenza pandemics that occurred in the past century (1918 H1N1, 1957 H2N2, 1968 H3N2, and 2009 H1N1) all originated in whole or in part from non-human reservoirs, and were associated with the genetic recombination of avian and other influenza viruses [1–4]. Due to the host species barrier, it is difficult for avian influenza viruses to spread from birds to humans directly, but they spread easily to pigs. Avian influenzas can become well-established and circulate through pig populations, for example the European swine H1N1 virus, which was introduced from wild ducks in late 1970s [5, 6]. Pigs are therefore proposed to be “mixing vessels” that facilitate interspecies transmission of avian viruses from the wild bird reservoir into humans and other mammals, and thus causes influenza pandemics in humans and other mammals [7–9].

For interspecies transmission of avian influenza virus to mammals, the virus needs to overcome two main host barriers: HA receptor-binding specificity; and the ability of the viral polymerase to replicate in mammalian cells [10, 11]. Pig cells have both human and avian influenza virus binding receptors on their surfaces [12, 13], allowing the first step of infection of pigs by bird and human influenza viruses [14]. The mechanism by which the RNA polymerase of avian influenza virus adapts in pigs is still unclear.

The influenza viral polymerase complex is composed of the PB2, PB1, and PA proteins. Together with the viral genome, and the nucleoprotein (NP), the polymerase forms viral ribonucleoprotein complex (vRNP) to carry out viral replication and transcription in infected cells [15, 16]. In general, the activity of the avian influenza viral polymerase is very limited in mammalian cells [17–23]. However, avian IAV can transmit to humans and other mammals by obtaining sufficient adaptive mutations (such as PB2 E627K) to facilitate sustained infection [24–30]. Surprisingly, the available evidence indicates that avian influenza viruses are capable of replicating and transmitting in pigs directly without mammal-adaptive mutations [31–42]. Epidemiological surveys of influenza A viruses in pigs show that avian influenza A viruses can naturally spill over into the pig population, and avian-origin viruses have been isolated from pigs worldwide [31–41]. Under laboratory conditions, avian IAV subtypes H1–H13 can infect and replicate in pigs and the pigs have different levels of susceptibility to the different viruses [42]. These observations suggest that certain unique cofactors in pigs, not present or different in other mammalian species, may play important roles in the replication of avian influenza viruses in pigs.

Many host factors are involved in mediating vRNP trafficking and promoting vRNP functions in infected cells [15, 16, 43, 44]. The proteins ANP32A and ANP32B, members of the acidic (leucine-rich) nuclear phosphoprotein 32 kDa (ANP32) protein family, were identified as crucial host factors that contribute to IAV polymerase activity [45–48]. Chicken ANP32A (chANP32A) has been previously reported as a host factor mediating species-specific influenza virus polymerase activity, because it harbors an exon duplication encoding a 33 amino acid insertion [46]. Avian species also differentially express other ANP32A splicing isoforms, either missing of the SUMO interaction motif-like sequence (SIM), or missing of the entire 33 amino acid insertion. These isoforms have differing abilities to support avian IAV polymerase [49–51]. Our and other studies have showed that ANP32A and ANP32B play a fundamental and critical role in viral polymerase activity. Most ANP32A and ANP32B proteins from different species contribute equally to IAV replication, with exception that chicken ANP32B is naturally inactive due to mutations at amino acid positions 129–130 [47, 52, 53]. Most mammalian ANP32A and ANP32B show poor support to avian influenza polymerase. Therefore, mammalian ANP32 proteins are supposed to be important host factors that restrict the replication of avian influenza viruses in mammals.

In this study, we found that swine ANP32A (swANP32A) showed stronger supporting activity to avian viral polymerase than did either swine ANP32B (swANP32B) or ANP32A and B proteins from other mammals. Molecular mapping revealed two mutations at amino acid positions 106 and 156, which determine this activity. An amino acid Valine in position 106, which is unique to swANP32A, combined with a Serine at position 156, is critical in supporting avian influenza polymerase activity and therefore virus infectivity in pig cells by enhancing the interaction between swANP32A and avian polymerase. Our work reveals the role of ANP32A by which pigs can serve as intermediate hosts and can allow avian influenza virus replication, and gives further insights into the evolution of mammalian ANP32A, that determines the host range of the avian influenza A virus.

## Results

### Avian influenza virus polymerase activity is stronger in the presence of ANP32A from pigs than that from other mammals

**Fig 1:**
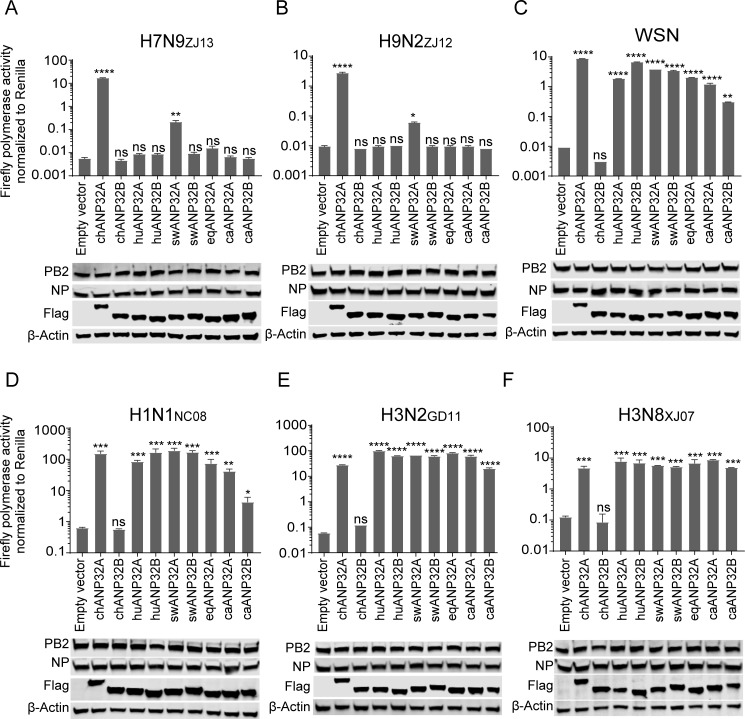
Species-dependent support of ANP32A or ANP32B for Influenza A viral replication. Vectors carrying 20 ng of ANP32A or ANP32B proteins, or empty vectors, were co-transfected into DKO cells, together with a minigenome reporter, a *Renilla* expression control, and influenza virus polymerases from either avian influenza H7N9_ZJ13_ (**A**) or H9N2_ZJ12_ (**B**); human influenza WSN (**C**); swine influenza H1N1_NC08_ (**D**); canine influenza H3N2_GD11_ (**E**); or equine influenza H3N8_XJ07_ (**F**). Luciferase activity was measured 24 h later. (Data are *Firefly* activity normalized to *Renilla*, Statistical differences between cells are labeled according to a one-way ANOVA followed by a Dunnett’s test; NS = not significant, *P < 0.05, **P < 0.01, ***P < 0.001, ****P < 0.0001. The results represent at least three independent experiments.) ch, chicken; hu, human; sw, swine; eq, equine; ca, canine.

Results from our previous work, and that of other labs, suggest that the host factors ANP32A and ANP32B play an important role in influenza A virus polymerase activity and virus replication in different animals [47, 48], with the truth that mammalian ANP32A&B proteins provide only limited support to avian influenza A viruses. However, until now the effects of the swine ANP32A and ANP32B proteins on avian influenza virus replication have remained largely unknown, although Long and his colleagues showed overexpression of pig ANP32A had no obvious effect on the polymerase activity of a turkey H5N1 virus in human 293T cells [46]. In our previous study, we developed an ANP32A&B double knockout 293T cell line (DKO) which has a large window for polymerase activity and provides a better system to illustrate ANP32 proteins’ function in supporting virus replication [47]. Here we cloned *ANP32* genes from different species, including chicken, human, pig, horse, and dog, and analyzed their ability to support polymerase activity of influenza A viruses from above mentioned species in DKO cells. The results showed that influenza A virus polymerases from all species tested were active in the presence of chicken ANP32A (chANP32A), but that none of the polymerases were active in the presence of chANP32B, which was consistent with previous studies [47] (Fig 1). Interestingly, ANP32A and ANP32B from most of the mammals studied (huANP32A, huANP32B, swANP32B, equine ANP32A (eqANP32A), canine ANP32A (caANP32A), and canine ANP32B (caANP32B)), showed only poor support to the polymerase activities of avian influenza viruses A/chicken/Zhejiang/DTID-ZJU01/2013 (H7N9_ZJ13_) [54] and A/chicken/Zhejiang/B2013/2012 (H9N2_ZJ12_) [55]. Surprisingly, swANP32A showed mild ability to support polymerase activities of H7N9_ZJ13_ and H9N2_ZJ12_ (Fig 1A and 1B). Mammalian ANP32 proteins showed the support ability to the replication of mammalian influenza A viruses, including human influenza virus A/WSN/1933 (WSN) [56] (Fig 1C), swine influenza virus A/swine/North Carolina/3793/08 (H1N1_NC08_) [57] (Fig 1D), H3N2 canine influenza virus A/canine/Guangdong/1/2011 (H3N2_GD12_) [58] (Fig 1E), and A/equine/Xinjiang/1/2007 (H3N8_XJ07_) [59] (Fig 1F). In addition, we noticed that some mammalian influenza viruses had lower activity in the presence of caANP32B than with caANP32A (Fig 1). These results indicated that, unlike other mammalian ANP32A&B proteins, the swANP32A has a stronger ability to support avian influenza virus polymerase.

### swANP32A has a stronger interaction with avian viral polymerase than does huANP32A

**Fig 2:**
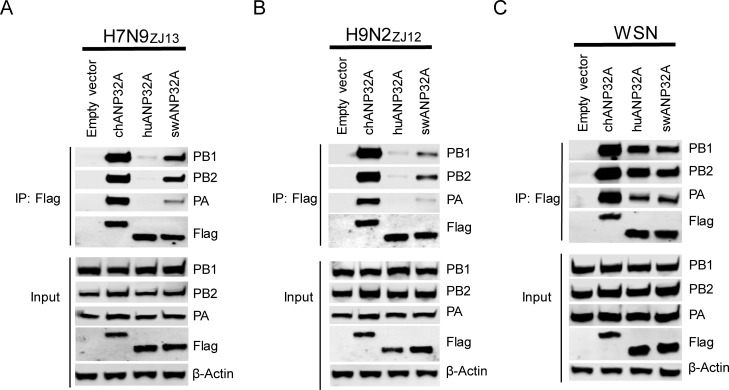
ANP32A proteins from different species interact with different polymerase trimeric complexes. DKO cells were transfected with different ANP32A (0.6μg) and polymerase plasmids (0.6μg PA, 1μg PB1, and 1μg PB2) from avian influenza viruses H7N9_ZJ13_ **(A)**, H9N2_ZJ12_ **(B)**, human influenza virus polymerase WSN **(C)**. The cells were lysed at 24 h post-transfection. Co-IP was performed using Anti-FLAG M2 Magnetic Beads, followed by Western blotting to detect the ANP32A and viral proteins by using specific antibodies: PA antibody (NBP2-42874, NOVUS), PB1 antibody (NBP2-42877, NOVUS), PB2 antibody (NBP2-42879, NOVUS), Anti-Flag antibody (F1804, SIGMA).

ANP32A proteins from birds, humans, and pigs showed differing levels of activity to support avian influenza polymerases in our study. It is thought that this effect on the activity of viral polymerases may be related to the polymerase complex binding with ANP32A protein. To investigate the binding capacity of ANP32A from different species to avian influenza virus polymerase, ANP32A constructs were co-transfected with the avian influenza polymerase into DKO cells. Coimmunoprecipitation results showed that chANP32A had a strong interaction with avian H7N9_ZJ13_ polymerase while huANP32A had only a weak interaction with H7N9_ZJ13_ polymerase (Fig 2A). Importantly, swANP32A was able to bind, albeit moderately, to the avian virus H7N9_ZJ13_ polymerase (Fig 2A). We obtained similar results using another avian influenza H9N2_ZJ12_ polymerase context (Fig 2B), and these interactions were RNA-independent (S1 Fig), suggesting that swANP32A showed support to avian influenza viral polymerase activity because of its direct interaction with the polymerase. ANP32A constructs were co-transfected with the human influenza WSN polymerase as controls, and huANP32A and swANP32A showed similar binding to WSN polymerase (Fig 2C), in agreement with the polymerase activity assay (Fig 1).

### Knockout of ANP32A in pig cells sharply reduced avian influenza viral RNA replication

**Fig 3:**
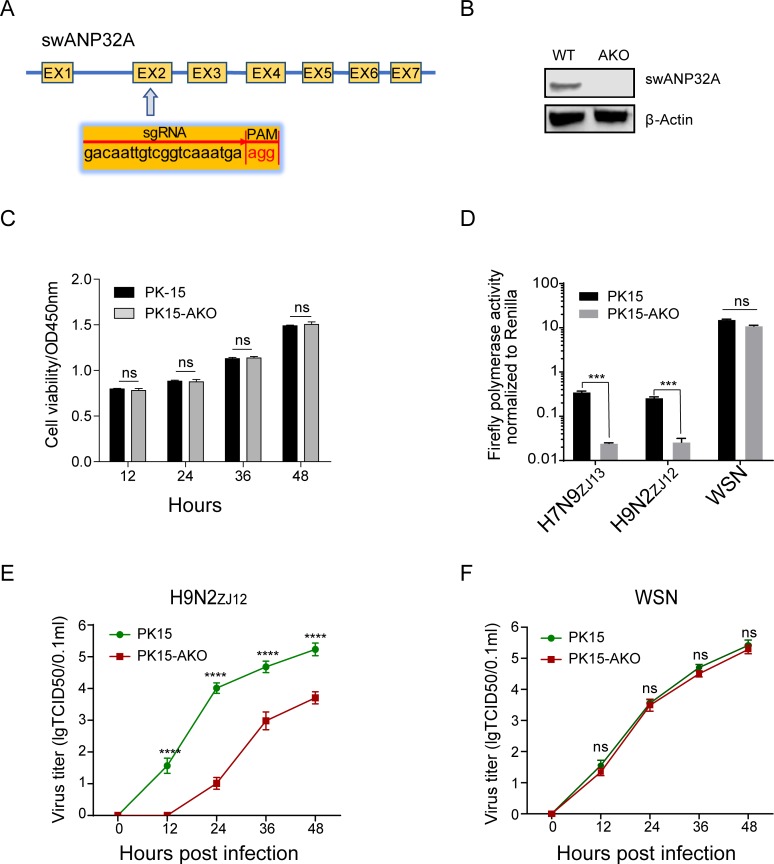
Knockout of ANP32A in pig cells sharply reduced avian influenza viral RNA replication. **(A)** The scheme of sgRNA used for knocking out the *swANP32A* gene. PK15 cells were transfected with pMJ920 vector (plasmid expressing eGFP and Cas9) and gRNAs to generate swANP32A knockout cells (PK15-AKO). **(B)** The endogenous proteins in wild type and knockout PK15 cells were identified by western blotting using antibodies against β-actin (sc-47778, Santa Cruz) and swANP32A (Rabbit Anti-PHAP1 polyclonal antibody, bs-6083R, Bioss). **(C)** Wildtype and AKO PK-15 cells were seeded in 96-well plate at 1x10^4 cells per well, 10 μL of CCK-8 reagents (meilunbio, MB4420) were added at the indicated times, and then the 96-well plate was incubated at 37°C for 1.5 h. The optical density (OD) values were measured by a microplate reader at OD450nm wavelength. **(D)** Wild type and knockout PK15 cells were transfected with *Firefly* minigenome reporter, *Renilla* expression control, together with polymerases from the avian influenza viruses H7N9_ZJ13_, or H9N2_ZJ12_, or the human influenza virus WSN. Luciferase activity was measured at 24 h post transfection. Data are *Firefly* activity normalized to *Renilla*. Statistical differences between cells are labeled according to a one-way ANOVA followed by a Dunnett’s test; NS = not significant, ***P < 0.001. **(E and F)** Wild type and knockout PK15 cells were infected with avian influenza virus H9N2_ZJ12_ and human influenza virus WSN at an MOI of 0.1, respectively. The supernatants were sampled at 0, 12, 24, 36, 48 h post infection and the virus titers were determined by means of endpoint titration in PK15 cells. (Statistical difference between groups were labeled, according to a one-way ANOVA followed by a Dunnett’s test; NS = not significant, ****P < 0.0001).

We have found that swANP32A, but not ANP32A proteins from other mammals, is unique in supporting avian influenza viral polymerase activity. In order to verify the effect of swANP32A on the replication and infection of avian influenza virus in pig cells, we first constructed ANP32A knockout pig kidney epithelial PK15 (referred to as PK15-AKO) cell line by using CRISPR-Cas9 system targeting the second exon of the *ANP32A* gene (Fig 3A). The PK15-AKO cell line was identified by Western blotting (Fig 3B). There was no difference in cell viability between PK15-AKO and wild type PK-15 cells detected by Cell Counting Kits-8 (CCK-8) (Fig 3C). The polymerases from the avian influenzas H7N9_ZJ13_ and H9N2_ZJ12_, and from human influenza WSN were transfected with pHH21-pgPolI-vLuc into PK15-AKO and wildtype PK15 cells, and polymerase activities were measured at 36h post transfection. The polymerase activities of both H7N9_ZJ13_ and H9N2_ZJ12_ were significantly reduced when swANP32A was absent, while the polymerase activity of WSN did not change whether or not swANP32A was present (Fig 3D). Additionally, the titer of avian H9N2_ZJ12_ virus was reduced dramatically in pig cells following knocking out of swANP32A (Fig 3E), showing that the polymerase was unable to function well without the swANP32A protein. As expected, human WSN virus exhibited similar growth kinetics in PK15-AKO cells and wild type PK15 cells (Fig 3F), indicating that endogenous swANP32B can support the replication of non-avian influenza A virus sufficiently.

### Mapping the sites in swANP32A key to its ability in supporting avian viral polymerase activity

**Fig 4:**
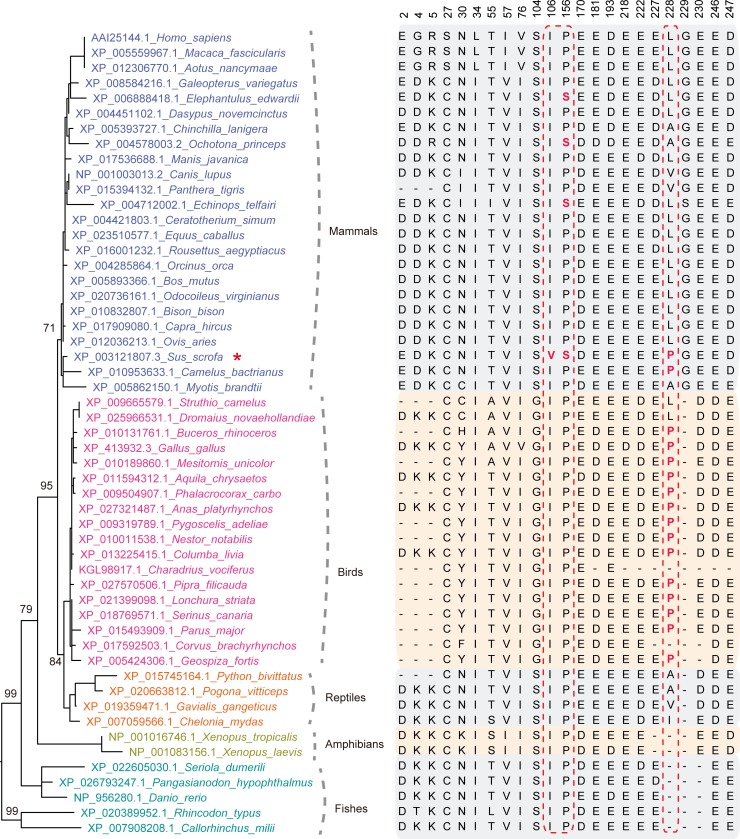
Phylogenetic analysis and amino acid sequence alignments of ANP32A proteins from different species. Bootstrap support values are indicated near the selected nodes. Right-hand columns display different amino acids in vertebrate ANP32A proteins.

ANP32A proteins have been found expressed in many species through the animal, plant and protist kingdoms, although not in yeast or other fungi [60]. These proteins range from 220 to 290 amino acid residues in length and are characterized by an N-terminal LRR domain and a C-terminal LCAR region. Sequence analysis indicated that ANP32A from pigs shared higher sequence identity with its counterparts from humans and other mammals than with those from birds (Fig 4). Phylogenetic analysis showed that ANP32A proteins from birds and mammals are from different evolutionary lineages (Fig 4).

**Fig 5:**
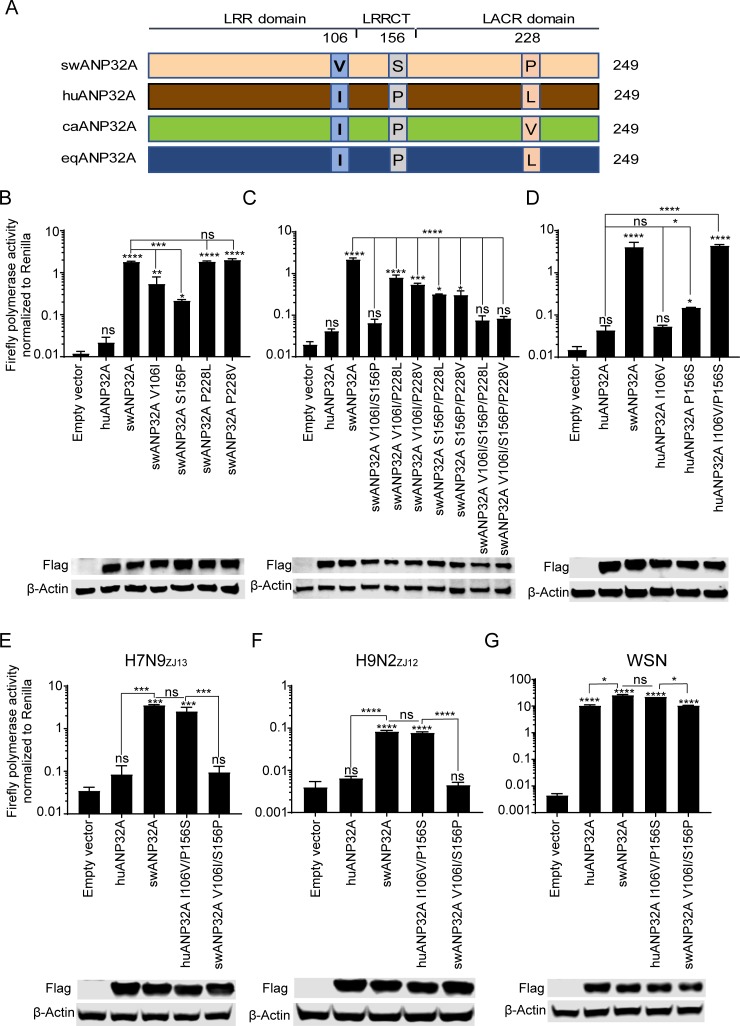
Mapping the unique sites of swANP32A responsible for the support for avian influenza viral replication. **(A)** Schematic of alignment from mammalian ANP32A amino acid sequences including human ANP32A (huANP32A), canine ANP32A (caANP32A), equine ANP32A (eqANP32A) and swine ANP32A (swANP32A). Three residues were annotated as there are mutations on swANP32A, at positions 106, 156, and 228. **(B, C)** The mutants of swANP32A were constructed by overlapping PCR. DKO cells were co-transfected with expression plasmids carrying PB1 (40 ng), PB2 (40 ng), PA (20 ng) and NP (80 ng) from H7N9_ZJ13_, together with 40 ng minigenome reporter and 10 ng *Renilla* luciferase expression plasmids (pRL-TK, as an internal control) in the presence of swANP32A or its mutants or empty vector. Cells were then lysed using passive lysis buffer and luciferase activity was measured at 24 h post transfection. **(D)** The mutants of huANP32A were co-transfected into DKO cells together with polymerase plasmids from H7N9_ZJ13_, plus a minigenome reporter and a *Renilla* luciferase control. Luciferase activity was analyzed as described above. **(E to G)** The mutants of huANP32A or swANP32A were co-transfected together with plasmids carrying the polymerases from H7N9_ZJ13_ (**E**), H9N2_ZJ12_ (**F**), or WSN (**G**). Luciferase activity was analyzed as described above. (B to G, data are *Firefly* activity normalized to *Renilla*. Statistical differences between cells are labeled according to a one-way ANOVA followed by a Dunnett’s test; NS = not significant, *P<0.05, **P < 0.01, ***P < 0.001, ****P < 0.0001).

The amino acid sequences of swANP32A, huANP32A, caANP32A, and eqANP32A were further analyzed. Sequence alignment showed that in three positions, positions 106, 156, and 228, swANP32A differed with those from other animals (Figs 4 and 5A). To investigate this further, single site mutations were introduced into swANP32A separately: V106I, S156P, P228L, and P228V. Then plasmids carrying these mutants and wild type swANP32A were separately co-transfected with the H7N9_ZJ13_ polymerase into DKO cells. The results showed that the V106I and S156P mutants slightly impaired the ability of swANP32A in supporting avian H7N9_ZJ13_ polymerase, while mutations at position 228, P228L and P228V, both showed no effect on the function of swANP32A (Fig 5B), indicating that single amino acid alternation was not able to change the ability of swANP32A in supporting avian influenza viral polymerase sufficiently.

Next, double- as well as triple-site mutations were generated to investigate the effect of these amino acid changes on the activity of the viral polymerase. The luciferase assay further suggested that the double-site mutation V106I/S156P could reduce polymerase activity completely, to the same extent as either using the huANP32A or an empty vector, indicating positive epistasis between 106V and 156S (Fig 5C). Meanwhile, the double-site mutations V106I/P228L and V106I/P228V could only partially decrease the activity of the viral polymerase, similar to the single mutation V106I. A similar phenotype was observed with the mutants S156P/P228L and S156P/P228V. Triple-site mutations V106I/S156P/P228L and V106I/S156P/P228V showed a similar effect to the double-site mutation V106I/S156P, reducing the activity of the viral polymerase fully (Fig 5C). These results indicated that both V106 and S156 residues, but not the P228 residue, play an important role in the species-specific activity of swANP32A to support avian influenza viral polymerase. The effect of these two amino acids, V106 and S156, was assessed by introducing reverse mutations in huANP32A. As expected, the double-site mutation I106V/P156S could allow avian H7N9_ZJ13_ polymerase to use huANP32A, while single-site mutants lead to slightly increased polymerase activities compared with wild type huANP32A (Fig 5D).

We further confirmed the effect of these two residues by using different influenza viral polymerases, including those from avian influenza H7N9_ZJ13_ (Fig 5E), H9N2_ZJ12_ (Fig 5F), and human influenza WSN (Fig 5G). The V106I/S156P mutation on swANP32A impaired its ability to support avian influenza H7N9_ZJ13_ and H9N2_ZJ12_ polymerase activity to the same extent as did huANP32A or an empty vector. However, the reverse I106V/P156S mutation on huANP32A enabled the avian influenza H7N9_ZJ13_ and H9N2_ZJ12_ polymerases to function, to the same extent as did swANP32A. Furthermore, WSN polymerase activity was retained in both these mutants (Fig 5G). Taken together, these results indicated that these two residues, 106V and 156S, are of great importance for swANP32A in supporting the activity of the avian viral polymerase in pigs.

### The key sites 106V/156S in swANP32A affect its interaction with viral polymerase and the synthesis of viral RNAs

**Fig 6:**
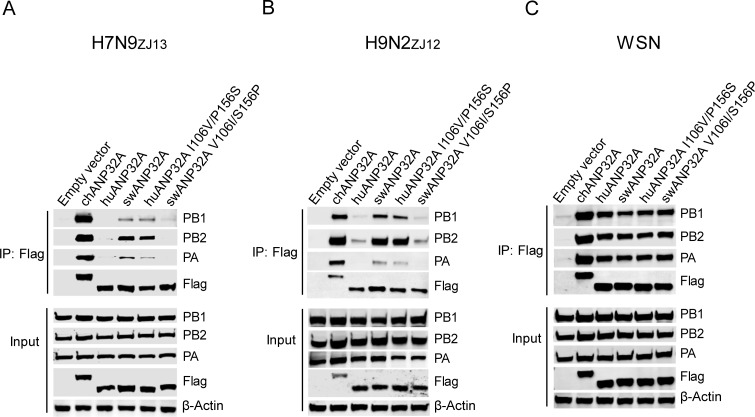
106V/156S sites of swANP32A are crucial for the interaction with avian polymerase trimeric complexes but not with mammalian polymerase. DKO cells were transfected with different ANP32A or mutants or empty vector (0.6μg), and plasmids carrying components of the viral polymerase (0.6μg PA, 1μg PB1, and 1μg PB2) from avian influenza virus H7N9_ZJ13_ **(A)**, H9N2_ZJ12_ **(B)**, or human influenza virus WSN **(C)**. The cells were lysed at 24 h post-transfection. Co-IP was performed using Anti-FLAG M2 Magnetic Beads, followed by Western blotting to detect the ANP32A and viral proteins by using specific antibodies, PA antibody (NBP2-42874, NOVUS), PB1 antibody (NBP2-42877, NOVUS), PB2 antibody (NBP2-42879, NOVUS), Anti-Flag antibody (F1804, SIGMA).

In order to confirm the importance of these two sites 106 and 156, a Co-IP experiment was performed to determine the interaction between avian influenza polymerase complex and ANP32A or its variants. We found that chANP32A had a strong interaction with avian influenza H7N9_ZJ13_ polymerase, with swANP32A able to bind H7N9_ZJ13_ polymerase moderately well, while huANP32A was unable to bind to avian influenza H7N9_ZJ13_ polymerase. The mutations at points 106 and 156 on swANP32A and huANP32A reversed the phenomenon, as swANP32A variant V106I/S156P lost the interaction with the H7N9_ZJ13_ polymerase, while huANP32A variant I106V/P156S had a moderate interaction with H7N9_ZJ13_, to the same extent as did swANP32A (Fig 6A). Similar results were obtained by using the polymerase from the avian H9N2_ZJ12_ influenza virus (Fig 6B). Meanwhile, the human influenza WSN viral polymerases showed a similar ability to bind huANP32A and swANP32A, and ANP32A mutants did not change the interaction of these proteins with the WSN polymerase (Fig 6C). The Co-IP results were consistent with the polymerase assay described above (Fig 5E–5G), indicating a special ability of swine ANP32A to support avian IAV replication which is enabled by the unique 106V/156S signature.

**Fig 7:**
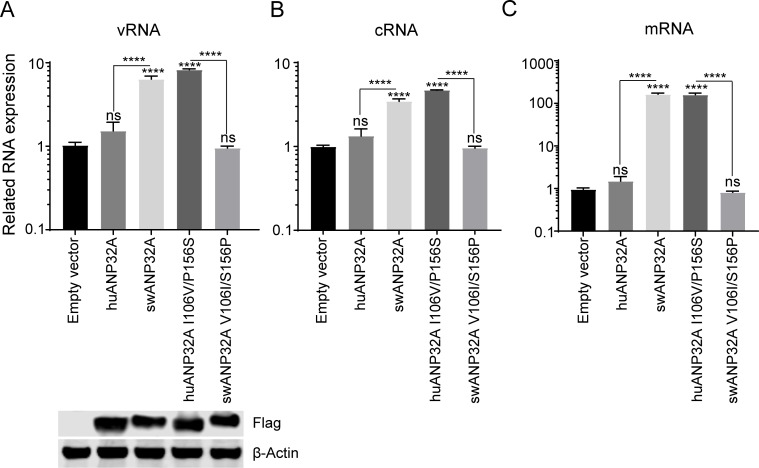
Effect of the ANP32A 106/156 sites on avian influenza viral polymerase transcription and replication. DKO cells were transfected with the minireplicon system of avian influenza virus H7N9_ZJ13_, and a minigenome reporter pHH21-huPolI-vLuc, together with different ANP32A mutants or empty vector. Total RNA of DKO cells was extracted at 24 h post-transfection and reverse transcription was performed, followed by quantitative PCR (qRT–PCR) for vRNA **(A)**, cRNA **(B),** and mRNA **(C)** from the luciferase gene. Luciferase RNA levels were normalized to the β-actin RNA level (means SD from three independent experiments. NS = not significant, ****P < 0.0001; all by one-way ANOVA followed by a Dunnett’s test). The expression of different ANP32A mutants were evaluated by Western blotting.

Influenza virus polymerase is responsible for influenza viral genome replication and transcription [61]. Our previous study has shown that ANP32A&B affect polymerase activity by regulating the synthesis of vRNA, cRNA and mRNA [45, 47]. Here, to test the effect of mutant ANP32A proteins on the synthesis of avian influenza viral RNA, the mutants and wild type ANP32A were co-transfected with the avian influenza H7N9_ZJ13_ polymerase together with a minigenome reporter plasmid pHH21-huPolI-vLuc, into DKO cells. Luciferase RNAs derived from the influenza polymerase were quantified using qRT-PCR. The results showed that the vRNA, cRNA, and mRNA synthesis were significantly increased in the presence of swANP32A but not huANP32A, while the mutations at positions 106 and 156 on swANP32A and huANP32A reversed the observed activities in both experiments (Fig 7).

In short, these results suggest that 106V/156S is a key signature on swANP32A that allows an interaction with the avian influenza viral polymerase, and the subsequent synthesis of viral RNAs.

### The key signature 106V/156S in swANP32A is responsible for its activity in supporting avian influenza virus infection in pig cells

**Fig 8:**
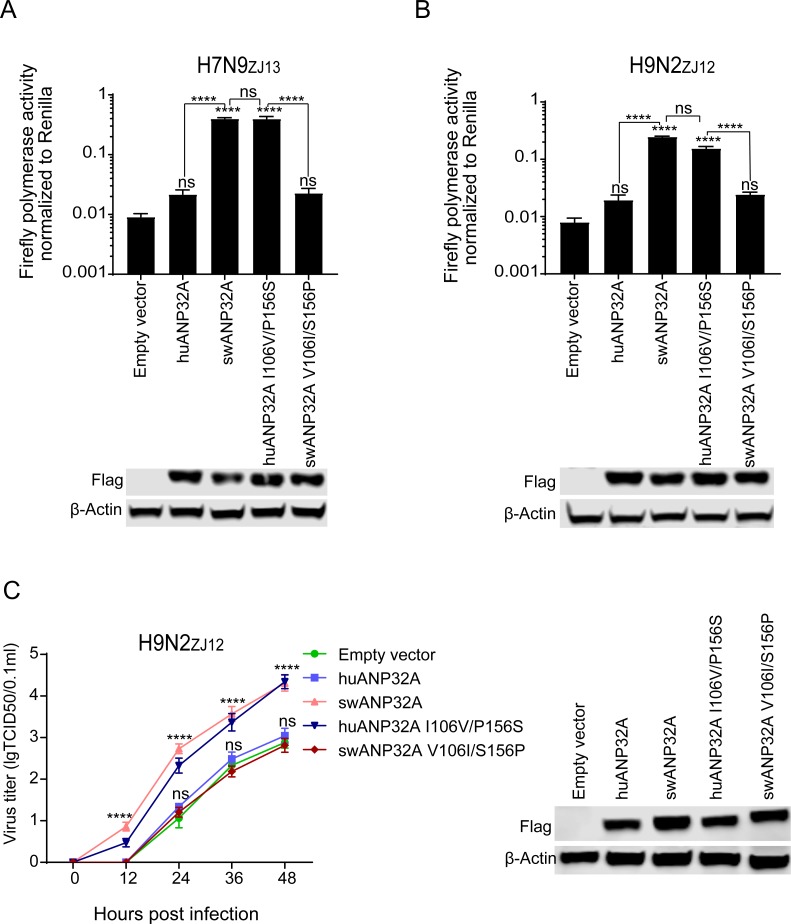
106V/156S sites of swANP32A are responsible for the infectivity of avian influenza virus in PK15 cells. Plasmids (200ng) carrying ANP32A or mutants, or empty vectors were co-transfected with plasmids carrying polymerase from H7N9_ZJ13_ **(A)** or H9N2_ZJ12_ **(B)**. Luciferase activity was assayed at 36 h after transfection. (Data are *Firefly* activity normalized to *Renilla*. Statistical differences between cells are labeled according to a one-way ANOVA followed by a Dunnett’s test; NS = not significant, ***P < 0.001, ****P < 0.0001). **(C)** PK15-AKO cells were transfected with 1μg ANP32A or mutants or empty vector. After 24 h, the cells were infected with avian influenza virus H9N2_ZJ12_ at an MOI of 0.1. The supernatants were collected at 0, 12, 24, 36, 48 h post infection and the virus titers in these supernatants were determined as above. The expressions of different ANP32A mutants were evaluated by Western blotting. (Statistical difference between cells were labeled, according to a one-way ANOVA followed by a Dunnett’s test; NS = not significant, ****P < 0.0001).

We next tested these different variants in the PK15-AKO cells. The reconstitution of swANP32A in the PK15-AKO cells restored the viral polymerase activities of H7N9_ZJ13_ and H9N2_ZJ12_, while the swANP32A variant V106I/S156P did not. Meanwhile, the reconstitution of huANP32A in the PK15-AKO cells could not recover the viral polymerase activity of H7N9_ZJ13_ and H9N2_ZJ12_, but huANP32A variant I106V/P156S could (Fig 8A and 8B), which indicated that avian influenza polymerase can recruit swANP32A to support its activity, due to the unique sites 106V/156S in this protein. Moreover, viral titer was enhanced by the expression of swANP32A or the huANP32A I106V/P156S variant in PK15-AKO cells but not by huANP32A or the swANP32A V106I/S156P variant (Fig 8C), suggesting that the sites 106V/156S are vital for the replication of the avian influenza virus.

In conclusion, we confirmed that avian influenza viral replication and polymerase activity can occur through interaction with swANP32A, and verified a unique site 106V/156S on swANP32A, but not in the ANP32A from other animals, that allows this interaction.

## Discussion

Influenza A viruses (IAVs) are zoonotic agents, capable of crossing the species barrier. The viral polymerase is a major determinant of host range [10, 11, 62]. Generally, avian influenza polymerase activity is heavily limited in mammalian cells. Pigs are considered to be important intermediate hosts for the transmission of avian influenza virus between birds and mammals, as they are susceptible to infection with both avian and human influenza viruses, and genetic re-assortment between avian, human, and swine influenza viruses can occur in pigs. Many subtypes of avian influenza viruses, including the highly pathogenic AIV (HPAIV) of the H5 subtype, have been isolated from pigs [63], suggesting that pigs have a special molecular mechanism which allows the infection and replication of avian influenza viruses.

ANP32 proteins, including ANP32A and ANP32B, are key functional co-factors affecting IAV polymerase adaption and activity, and are also important in the replication of other viruses such as HIV-1 [64]. ANP32A and ANP32B proteins from most mammals do not support avian influenza polymerase activity, providing a host barrier against avian influenza virus interspecies transmission. Furthermore, huANP32A has been proposed as being involved in the emergence of the adaptive mutation PB2 E627K in the H7N9 virus strain [65]. In this study, we show that swANP32A, as well as other ANP32A from different species including human, dog, and horse, are universally capable of supporting influenza viral polymerases from humans, pigs, dogs, and horses. Interestingly, compared with human and other mammalian ANP32As, which do not support avian polymerase activity, swANP32A shows a mild support to avian influenza virus polymerase activity. Knockout of swANP32A in pig PK15 cells significantly reduces the infection of avian influenza virus. These observations agree with the fact that: 1) avian influenza viruses can replicate in birds and pigs directly, but not in humans or other mammals without mammal-adaptive mutations; 2) pigs are thought to be intermediate hosts as they are susceptible to infection with both avian and mammalian influenza viruses.

Only a few studies have focused on the replication ability of avian IAV polymerase in pig cells. Moncorge and his coauthors observed that avian IAV polymerase could not work well in pig NPTr or human 293T cells, compared with its activity in avian DF-1 cells, which was a controversial result due to the fact that the pigs can be infected by some avian viruses. This may be due to an imperfect detection system (lacking the control luciferase) and varies transfection efficiency in different kinds of cells as mentioned by the authors [66]. The ANP32A and ANP32B double knockout cells provide a clear background to study the IAV viral polymerase activity. Without ANP32A&B, the viral polymerase activity reduced around 10000 fold compared to the control, indicating the key role of ANP32A&B in supporting viral polymerase activity [47]. Reconstitution of swANP32A, but not of other mammalian ANP32 proteins, enhanced chicken influenza virus polymerase activity in the DKO cells (Fig 1). Knockout of ANP32A in pig PK15 cells reduced avian viral replication and viral polymerase activities (Fig 3). These results confirmed the function of swANP32A in supporting avian IAV replication in pig cells.

**Fig 9:**
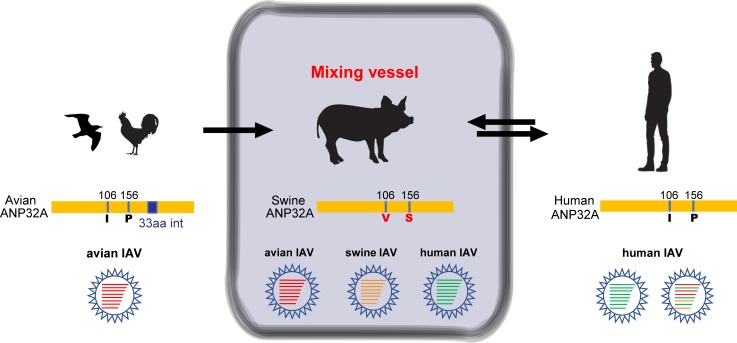
Proposed model of ANP32 proteins as “the key” for the inter-species transmission of avian influenza A virus in different species. AIVs can replicate and transmit naturally in birds. Pigs are thought to play an important role in influenza virus transmission and triple-reassortant virus generation, because they are susceptible to both avian and mammalian influenza viruses, and different species of influenza viruses can therefore genetically recombine in pigs, producing new strains that can transmit to humans. Avian ANP32A can support the replication of the avian influenza virus due to an additional 33-aa insertion; huANP32A only can support the polymerase activity of mammalian or mammal-adapted (e.g. PB2 E627K, or D701N) viruses, but not that of avian viruses); as swANP32A contains a unique active site 106V/156S, it can support the replication of not only all mammalian influenza viruses, but also avian influenza viruses. The co-infection of two or more virus strains in pig providing more chances to produce new recombinant strains, which can spread to other mammals such as humans.

Phylogenetic analysis of ANP32A proteins suggested that avian and mammalian ANP32A formed different evolutionary linages (Fig 4). ANP32A proteins from different species have relatively conserved structure with scattered amino acid sequences substitutions (S2 Fig). However, all ANP32A proteins have a hyper variable region at the N terminal. Differentially spliced avian ANP32A transcripts encode isoforms harboring a 33-aa or a 29-aa insertion between the LRRs and LACR regions, which are distinct from mammalian proteins [50, 51]. The avian ANP32As with the 33-aa or 29-aa insertion have stronger binding abilities to IAV polymerase than human ANP32A, and these binding abilities are independent of viral polymerase E627K adaptation [49, 50]. Sequence analysis showed that swANP32A shares high sequence identity with its counterparts from human and other mammals, with a few substitutions. Here we revealed that swine ANP32A, unlike ANP32A proteins from other mammals, has a unique 106V/156S sequence signature, which supports the replication of avian influenza viruses in pig cells. The 106V is specific in pigs among mammalian species, and the positive epistasis between 106V and 156S provides a molecular basis for avian IAV replication in pigs. Of note, swANP32A showed stronger binding ability to IAV polymerase than did huANP32A, but weaker than did chANP32A. Furthermore, the 106V/156S determined the binding ability of swANP32A (Fig 6). This is the first time that the molecular mechanism by which pigs can act as the “mixing vessel” for the inter-species transmission of influenza A virus (as showed in a schema diagram Fig 9) has been suggested.

To date, some key sites in ANP32 proteins have been identified. Avian ANP32A has an additional 33 amino acid insertion located in the LRRCT region, which is crucial for its ability to support AIV polymerase activity [46]. Deletion of a SUMO interaction motif (SIM)-like sequence or an alternative splicing form of avian ANP32A with a 29 amino acid insertion has been shown to slightly reduce viral RNA replication [49–51]. Our previous work revealed that influenza A virus polymerase activity and host range is closely linked to host ANP32A and ANP32B proteins [47]. Furthermore, the key sites 129N/130D, which are located in the LRRCT region, are vital for the interaction of ANP32 proteins in animal cells with viral polymerase [47]. In this study, we found that the unique sites 106V and 156S in host swANP32A are crucial for its activity to support avian IAV replication. The 106V/156S signature is to date the only site in the mammalian ANP32 protein which is known to be important for the avian influenza virus. It has been confirmed that these sites regulate the polymerase activity by mediating the interaction between ANP32 proteins and the polymerase complex. Therefore, we speculate that the mutations of these key sites in ANP32A may alter its conformation and disrupt its interaction with the viral polymerase complex. However, the structure of ANP32A is difficult to be parsed because of its low complexity acidic region. Further work is needed to better understand the molecular mechanism of the interaction of this protein with the viral polymerase, and to provide new molecular targets for the design of novel influenza virus control strategies.

In summary, we provide evidence in this study that species differences in cellular ANP32A determine avian influenza A virus polymerase host restriction. We also identify a unique key site, 106V, in swine ANP32A but not in ANP32A from any other mammals or bird studied, which, together with 156S, enables an interaction between swine ANP32A and avian viral polymerase, promoting viral replication. Our work may help to understand the interplay between mammalian ANP32A proteins and avian viral polymerase complex and the molecular mechanism by which pigs are “mixing vessels” for avian and mammalian influenza viruses.

## Materials and methods

### Ethics statements

The 9-day-old embryonated chicken eggs were obtained from Harbin Veterinary Research Institute of the Chinese Academy of Agricultural Sciences. The protocols for influenza virus propagation on chicken embryos were reviewed and approved by the Animal Ethics Committee of Harbin Veterinary Research Institute of the Chinese Academy of Agricultural Sciences (SYXK (Hei) 2017–009).

### Cells, viruses, and plasmids

Human embryonic kidney ANP32A and ANP32B double-knockout 293T cell lines (DKO cells, described previously) and pig kidney epithelial (PK15, ATCC CCL-33) cells were cultured in the growth medium (Dulbecco’s modified Eagle’s medium (DMEM, Hyclone) with 10% fetal bovine serum (FBS; Sigma) and 1% Penicillin-Streptomycin (Gibco)), and kept at 37°C in a 5% CO_2_ atmosphere. Plasmids carrying the polymerase from influenza A viruses from different species were used in this study, and included those from: H1N1 human influenza virus A/WSN/1933 (WSN, kindly provided by Dr Yoshihiro Kawaoka); H7N9 chicken influenza virus A/chicken/Zhejiang/DTID-ZJU01/2013(H7N9_ZJ13_, kindly provided by Dr. Hualan Chen); H1N1 swine influenza virus A/swine/North Carolina/3793/08 (H1N1_NC08_, kindly provided by Dr Feng Li from South Dakota State University); H3N2 canine influenza virus A/canine/Guangdong/1/2011 (H3N2_GD12_, kindly provided by Dr Shoujun Li); H9N2 avian influenza virus A/chicken/Zhejiang/B2013/2012 (H9N2_ZJ12_, kindly provided by Dr Zejun Li); and A/equine/Xinjiang/1/2007 (H3N8_XJ07_, kept in our lab). pCAGGS-ANP32 plasmids were generated by gene synthesis (Synbio technologies, China) based on the sequences retrieved from GenBank, and included: chicken ANP32A (chANP32A, *Gallus gallus*, XM_413932.5, XP_413932.3); human ANP32A (huANP32A, *Homo sapiens*, NM_006305.3, NP_006296.1); swine ANP32A (swANP32A, *Sus scrofa*, XM_003121759.6, XP_003121807.3); equine ANP32A (eqANP32A, *Equus caballus*, XM_001495810.5, XP_001495860.2); canine ANP32A (caANP32A, *Canis lupus familiaris*, NM_001003013.2, NP_001003013.2); chicken ANP32B (chANP32B, *Gallus gallus*, NM_001030934.1, NP_001026105.1); human ANP32B (huANP32B, *Homo sapiens*, NM_006401.2, NP_006392.1); swine ANP32B (swANP32B, *Sus scrofa*, XM_021066477.1, XP_020922136.1); and canine ANP32B (caANP32B, *Canis lupus familiaris*, XM_022425638.1, XP_022281346.1). Mutants of these plasmids were generated by overlapping PCR and were identified by DNA sequencing.

### Knockout cell lines

PK15 swANP32A-knockout (named PK15-AKO) cell lines were generated by CRISPR/Cas9 system, as described previously [47]. Briefly, the gRNAs for *swANP32A* were designed online (http://crispr.mit.edu/) [67] and inserted into the pMD18-T vector, which contains the U6 promoter, a guide RNA scaffold, and U6 termination signal sequence. Plasmid pMJ920, containing Cas9-eGFP, was a gift from Jennifer Doudna (Addgene plasmid # 42234) [68]. PK15 cells were seeded in a 6-well plate and transfected with 1.0 μg pMJ920 plasmids plus 1.0 μg gRNA expression plasmids by using Lipofectamine LTX Reagent with PLUS Reagent (Invitrogen, Cat. 15338100) following the manufacturers’ protocols. Then GFP-positive cells were sorted by fluorescence-activated cell sorting (FACS) at 48h post-transfection, and positive knockout cell clones were then identified by western blotting. The cell viabilities of wildtype and AKO PK-15 cells were measured by Cell Counting Kits-8 (CCK-8, meilunbio, MB4420) following the instructions.

### Polymerase assay

To test the effect of various ANP32 proteins on activity of polymerases from different viruses, DKO cells were seeded in 12-well plates and transfected with plasmids carrying PB1 (80 ng), PB2 (80 ng), PA (40 ng) and NP (160 ng), together with 80 ng minigenome reporter (pHH21-huPolI-vLuc, described previously) [47] and 10 ng *Renilla* luciferase expression plasmids (pRL-TK, kindly provided by Dr Luban), using Lipofectamine 2000 transfection reagent following the manufacturers’ instructions. Cells were lysed with passive lysis buffer (Promega) at 24 h after transfection, and *Firefly* and *Renilla* luciferase activities were measured using a commercial kit (Dual-luciferase kit, Promega) with Centro XS LB 960 luminometer (Berthold technologies). The expression levels of polymerase PB2 and NP in different groups were assessed by western blotting using specific antibodies.

To analyze the activity of the influenza viral polymerase in PK15 cells, a reporter plasmid pHH21-pgPolI-vLuc containing swine RNA polymerase I promoter was constructed, based on the plasmid pHH21-huPolI-vLuc. PK15 cells were used to extract swine genomic DNAs by QlAamp DNA Mini Kit (QIAGEN, Germany). The swine RNA polymerase I promoter sequence was then amplified from genomic DNA by using primer pairs, based on a previous study [66]. PCR fragment was then inserted into the pHH21-huPolI-vLuc by infusion PCR. To determine the function of ANP32 proteins, PK15 cells and PK15-AKO cells in 12-well plates were transfected with plasmids of the PB1 (200 ng), PB2 (200 ng), PA (100ng) and NP (400 ng), together with 200 ng pHH21-pgPolI-vLuc and 20 ng pRL-TK, using Lipofectamine LTX Reagent with PLUS Reagent. Cells were lysed with 100ul of passive lysis buffer (Promega) at 36 h after transfection, and *Firefly* and *Renilla* luciferase activities were measured.

### Quantification of viral RNAs by real time PCR

This assay was performed as described previously [47]. Total RNA from DKO cells was extracted using a RNeasy mini kit (Qiagen) according to the protocol. The first strand cDNA derived from *Firefly* luciferase RNAs driven by influenza polymerase was amplified by a Reverse Transcription Kit (Cat.RR047A). Specific primers were then used in the RT reaction, as follows: 5’- CATTTCGCAGCCTACCGTGGTGTT-3’ for the *Firefly* luciferase vRNA; 5’-AGTAGAAACAAGGGTG-3’ for the *Firefly* luciferase cRNA; oligo-dT20 for the *Firefly* luciferase mRNA. The cDNA samples were quantified using real-time PCR with specific primers F (5’-GATTACCAGGGATTTCAGTCG-3’) and R (5’-GACACCTTTAGGCAGACCAG-3’) using SYBR Premix Ex Taq II (Tli RNaseH Plus) (TaKaRa Cat: RR820A). Fold change in levels of RNA was calculated using double-standard curve methods and with β-actin as an internal control.

### Influenza A virus infection

Avian influenza virus H9N2_ZJ12_ and human influenza virus WSN were propagated in 9-day-old specific pathogen-free (SPF) embryonated chicken eggs as described previously [47] and used to infect PK15 cells. Wild type PK15 cells and PK15-AKO cells were cultured separately in 6-well plates and infected at a multiplicity of infection (MOI) of 0.1 for 1 h, washed twice with PBS, and then maintained at 37°C in DMEM containing 0.3% BSA and tosylsulfonyl phenylalanyl chloromethyl ketone (TPCK)-trypsin (Sigma) at 1μg/ml. At the indicated time points, the culture supernatant was harvested. Viral titers in PK15 cells were determined using the method of Reed and Muench [69]. To determine the function of ANP32 proteins, PK15-AKO cells were cultured in 6-well plates and transfected with 1μg of either different ANP32A or mutants or empty vector. Twenty-four hours later, cells were infected with H9N2_ZJ12_ virus as described above. The growth data shown are the average results of three independent experiments.

### Immunoprecipitation assay

DKO cells were transfected with the indicated plasmids, and cells were lysed in lysis buffer (50 mM Hepes-NaOH [pH 7.9], 100 mM NaCl, 50 mM KCl, 0.25% NP-40, and 1 mM DTT) at 24h post-transfection, then centrifuged at 13,000× g and 4°C for 10 min. The lysates were incubated with Anti-FLAG M2 Magnetic Beads (SIGMA-ALDRICH, M8823) at 4°C for 2h. Immunocomplexes were eluted using a 3X Flag peptide and boiled in the SDS-PAGE loading buffer, subjected to SDS-PAGE and analyzed by western blotting. Signals were detected using a LI-COR Odyssey Imaging System (LI-COR, Lincoln, NE, USA). The cell samples were also digested by RNase A/T1 Mix (Thermo Scientific, EN0551) to confirm whether the interactions between ANP32 proteins and viral polymerase were RNA dependent. This experiment was carried out using the following primary antibodies: Influenza A Virus PA antibody (NBP2-42874, NOVUS), Influenza A Virus PB1 antibody (NBP2-42877, NOVUS), Influenza A Virus PB2 antibody (NBP2-42879, NOVUS), Anti-Flag antibody (F1804, SIGMA).

### Phylogenetic analysis and sequence alignment

We used the BLASTP algorithm to retrieve ANP32A protein sequences from 53 vertebrates (24 mammals, 18 birds, 4 reptiles, 2 amphibians, and 5 fishes) with human ANP32A protein as the query. ANP32A proteins were aligned using MAFFT v7.271 [70], and the alignment was then manually refined. The best fitting substitution model (JTT+F+G4) was selected using ModelFinder [71]. Phylogenetic analysis was performed using a maximum-likelihood method implemented in IQ-TREE v1.6.8 [72]. The node supports were evaluated using an ultrafast bootstrap method with 1,000 replicates [72]. To demonstrate the variability of ANP32A proteins from different species, including chicken, turkey, duck, zebra finch, ostrich, human, pig, dog, horse, mouse, and ferret, the online software WebLogo (http://weblogo.berkeley.edu/logo.cgi) was used to construct WebLogo diagrams.

### Statistics

Data analysis was performed in GraphPad Prism, version 7 (GraphPad Software, USA). Statistical significance was assessed using One-way ANOVA followed by a Dunnett’s post-test. Error bars represent the standard deviation (SD) or the standard error of the mean (SEM), as indicated in figure legends. NS, not significant (p>0.05), *p<0.05, **p<0.01, ***p<0.001, ****p<0.0001. All the experiments were performed independently at least three times.

## Supporting information

S1 Fig The interactions between ANP32A proteins and avian influenza polymerase were RNA-independent. **(A)** DKO cells were transfected with different ANP32A (0.6μg) and polymerase plasmids (0.6μg PA, 1μg PB1, and 1μg PB2) from avian influenza viruses H7N9_ZJ13_. The cells were lysed at 24 h post-transfection. Cell lysates with or without treatment with 2μL RNase A/T1 Mix (Thermo Scientific, EN0551) were incubated with Flag beads overnight at 4°C. Immunocomplexes were eluted using a 3X Flag peptide and boiled in the SDS-PAGE loading buffer, subjected to SDS-PAGE and analyzed by western blotting. **(B)** The cellular RNA extracted from equal number of cells used in the IP assay was digested with or without the treatment with 2μL RNase A/T1 Mix overnight at 4°C. Then the samples were assessed by 1.5% agarose gel electrophoresis.
(EPS)

S2 Fig WebLogo presentation of the variability of amino acid sequences of ANP32A from different species. The height of each stack corresponds to the level of nucleotide conservation at that position. When the nucleotide is invariant, only one letter is shown; when the nucleotide is variable, the most common substitutions are noted. The figure depicts the key sites involved in supporting the influenza viral polymerase activity. The universal site 129/130 is indicated with red diamonds, the unique sites 106 and 156 are indicated with red circles, and the 33-aa insertion, including SLS (SIM-like sequence) and 27-aa repeat, is labeled in line.
(TIF)
