# Supplementary figures and images for: A unique feature of swine ANP32A provides susceptibility to avian influenza virus infection in pigs
Source: PLoS Pathog. 2020 Feb 21;16(2):e1008330. doi: 10.1371/journal.ppat.1008330 (PMC7055917; doi:10.1371/journal.ppat.1008330)

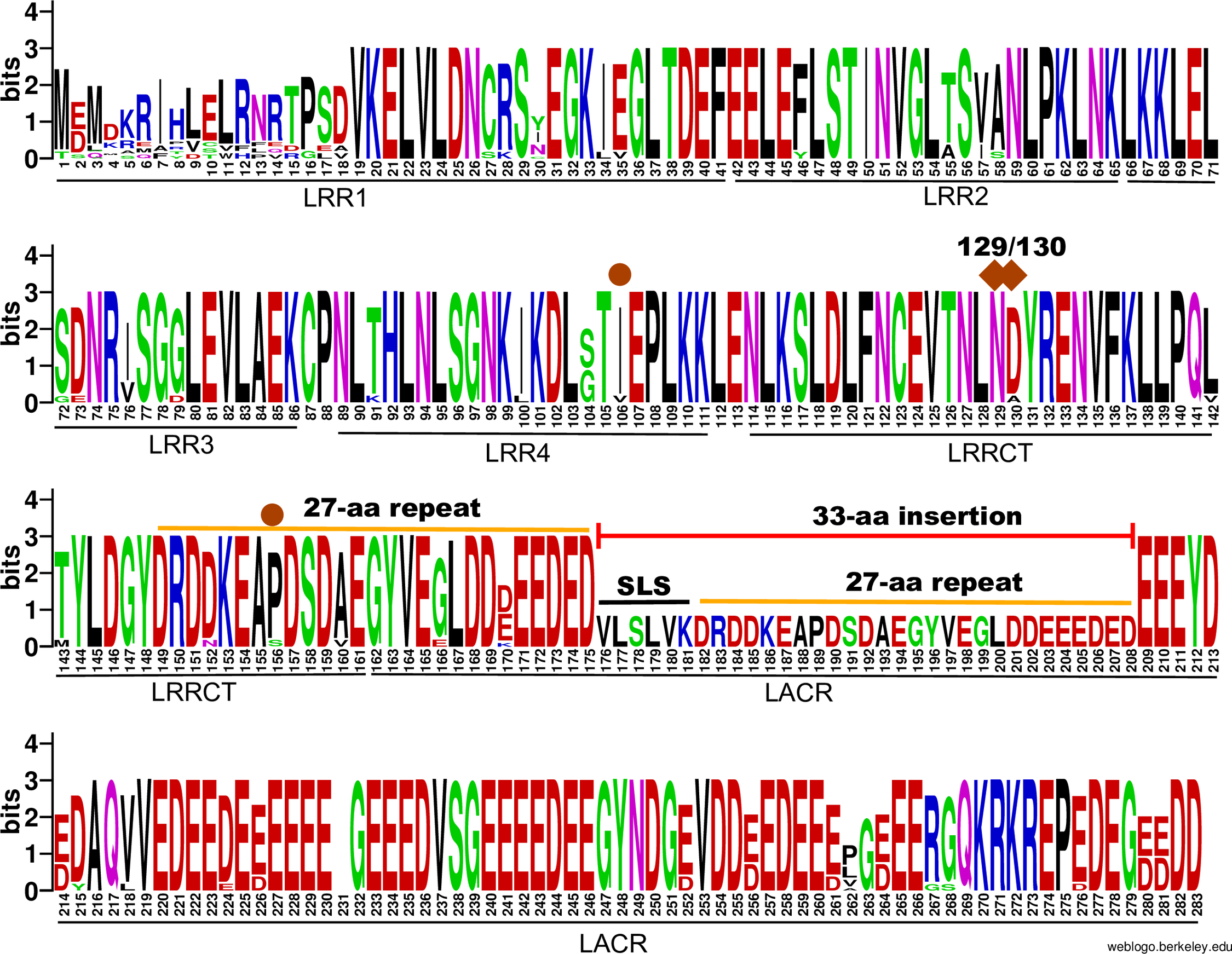

Supplement: S2 Fig — The height of each stack corresponds to the level of nucleotide conservation at that position. When the nucleotide is invariant, only one letter is shown; when the nucleotide is variable, the most common substitutions are noted. The figure depicts the key sites involved in supporting the influenza viral polymerase activity. The universal site 129/130 is indicated with red diamonds, the unique sites 106 and 156 are indicated with red circles, and the 33-aa insertion, including SLS (SIM-like sequence) and 27-aa repeat, is labeled in line. (TIF) [file ppat.1008330.s002.tif]
